# Hand fracture epidemiology and etiology in children—time trends in Malmö, Sweden, during six decades

**DOI:** 10.1186/s13018-019-1248-0

**Published:** 2019-07-12

**Authors:** Vasileios Lempesis, Björn E. Rosengren, Lennart Landin, Carl Johan Tiderius, Magnus K. Karlsson

**Affiliations:** Clinical and Molecular Osteoporosis Research Unit, Department of Clinical Sciences and Orthopedics, Lund University, Skåne University Hospital, SE-205 02 Malmö, Sweden

**Keywords:** Boy, Girl, Fractures, Epidemiology, Time trends

## Abstract

**Background:**

The aim of this study was to describe hand fracture epidemiology/etiology in city children and describe time trend during six decades.

**Patients and methods:**

A single hospital serves the entire city population of 271,271 (year 2005). Through the hospital medical and radiological archives, we collected epidemiology and etiology data concerning pediatric (age < 16 years) hand fractures in city residents, treated during 2005–2006. We compared these data to previously collected data in in the same city during 12 evaluated periods from 1950/1955 to 1993–1994. We present period-specific crude and age- and gender-adjusted fracture incidence rates and group differences as incidence rate ratios (RR) with 95% confidence intervals (95% CI).

**Results:**

In 2005–2006, we identified 414 hand fractures (303 in boys and 111 in girls), 247 phalangeal fractures (60% of all hand fractures), 140 metacarpal/carpal fractures (except the scaphoid bone) (34%), and 27 scaphoid fractures (6%). The crude hand fracture rate in children was 448/100,000 person years (639/100,000 in boys and 247/100,000 in girls), with a 2.5 times higher age-adjusted incidence in boys than in girls. Compared to 1950/1955, the age and gender-adjusted hand fracture incidence was twice as high in 2005–2006 and more than twice as high in 1976–1979. Compared to 1976–1979, we found no significant difference in the age and gender-adjusted hand fracture incidence in 2005–2006. In 2005–2006, sports injuries explained 42%, fights 20%, and traffic accidents 13% of the hand fractures. In 1950/1955, sports injuries explained 27% of fractures, fights 10%, and traffic accidents 21%.

**Conclusions:**

The incidence of hand fractures in children was more than twice as high in the end of the 1970s compared to the 1950s, where after no significant change could be found. Also, fracture etiology has changed. New studies are needed, to adequately allocate health care resources and identify new fracture prone activities suitable for preventive measures.

**Level of evidence:**

III

**Electronic supplementary material:**

The online version of this article (10.1186/s13018-019-1248-0) contains supplementary material, which is available to authorized users.

## Background

The hand is one of the most commonly injured anatomic regions, accounting for 29% of pediatric visits in emergency departments [1, 2]. Around one fourth of those patients have a fracture [3, 4], with the hand being the second most fractured region, surpassed only by the distal forearm [5–9]. However, there are gender differences in hand fracture occurrence, as well as differences in age incidences, with boys having higher incidences than girls and girls having an earlier peak incidence than boys [6, 8]. As time trends in fracture epidemiology have changed in boys and in girls during recent decades [3, 6, 7, 9–18], there is a need for updated pediatric fracture epidemiology. This also accounts for etiology data, in order to identify new fracture prone activities in need of prevention and evaluate the effect of already existing fracture prevention strategies.

The aim of this study is to describe hand fracture epidemiology/etiology in children in 2005–2006 and with use of previously published data from the same area [6, 9], evaluate time trends in age- and gender-standardized fracture incidences from 1950/1955 to 2005–2006.

## Patients and methods

Our city is located in the southern part of the country with a population of 276,244 inhabitants (46,429 < 16 years of age) in year 2006 [19]. One hospital provides trauma care for the entire city and this hospital archives store all medical charts, referrals, radiographs, and reports since a century [20], making it possible to re-evaluate former patients. Radiographs were until the year 2001, sorted according to anatomical region, year of injury, and diagnosis. Researchers have utilized this archive to describe pediatric fracture epidemiology in city residents < 16 years during the years 1950, 1955, 1960, 1965, 1970, 1975–1979 [6], and 1993–1994 [9]. These studies have reported changes in crude hand fracture incidences [6] and crude overall fracture incidences [9], however, without adjusting for changes in demography within the population at risk.

In 2001, the radiographic archive was replaced by a digital archive. This system includes radiographs performed within the healthcare system in the entire southern part of the country, including the Malmo city hospital. The radiographs are now classified according to each specific patient (through a unique 10-digit personal identity number), diagnosis, and anatomical location. Pediatric fracture cases 2005–2006 were therefore identified through this classification in the digital in- and outpatient diagnosis records at the Emergency Department, Departments of Orthopedics, Hand Surgery, and Otorhinolaryngology. We included records that fulfilled the following criteria: (i) ICD-10 fracture diagnosis: S02.3–S02.4, S02.6–S02.9, S12.0–S12.2, S12.7, S22.0, S32.0–S32.8, S42.0–S42.9, S52.0–S52.9, S62.0–S62.8, S72.0–S72.9, S82.0–S82.9, and S92.0–S92.9; (ii) age < 16 years at the time of the incident fracture; and (iii) city resident at the time of the injury.

We identified 4459 visits with a fracture diagnosis during 2005–2006 and 1548 with a hand fracture diagnosis (ICD-10 code S62.0-S62.8). To verify hand fractures, we reviewed medical charts, referrals, radiographic reports, and radiographs of all fracture visits. This enabled us to exclude all follow-up visits after an index fracture visit, thus avoiding double counting of a fracture. Patients who received emergency treatment in other hospitals are in our country, as standard, referred to the home hospital for follow-up evaluation. Such fractures were then captured in the hospital archives.

We collected pediatric fracture data from 2005 to 2006, following the same protocol as in the previous studies [6, 9]. Thus, we included data on patient age and gender, number of fractures, date of the fracture, fractured region/regions, and fracture etiology. During the years 2005–2006, we could determine the etiology in 70% of the cases compared to 40% during the years 1950/1955.

Multiple phalangeal fractures, multiple metacarpal fractures, and combinations of carpal and metacarpal fractures were registered as separate fractures. This was done to provide detailed information about the anatomical distribution of hand fractures and, by this, facilitate comparisons with other single center reports that register multiple metacarpal and phalangeal fractures separately [4, 12, 13, 17, 18, 21–26]. To compare the current data to the previously collected data from the same city, we recorded fracture events according to the previous classification protocol [6, 9], that is with three different groups: (i) fractures of the phalanges of the digits, (ii) fractures of the metacarpals or carpal bones, and (iii) fractures of the scaphoid. Multiple fractures in phalangeal, metacarpal, and carpal bones sustained at the same event were in this classification regarded as a single fracture, while multiple fractures on the same patient, bilateral fractures, and new fractures of an already fractured bone were classified as independent fractures [6, 9]. As in previous studies [6, 9], we did not include patients with traumatic amputations.

To validate the new fracture ascertainment system, one author (VL) performed a search in the digital radiological archive for all pediatric skeletal radiographs, independent on the reason for the referral, at the radiology department of the hospital from January 1, 2005, to February 28, 2005. The author reviewed all radiographs and then identified 103 pediatric fractures sustained in city residents. The same researcher then conducted a second search by the use of the same search criteria in the digital in- and outpatient diagnosis record archive. This second search also identified 103 fractures. One hundred fractures were identified by both methods; each method alone identified 103 fractures, while the two methods combined identified 106 fractures. Each method thereby missed three fractures, a miscalculation rate of 3%.

For statistical calculations, we organized hand fracture data in six periods (1950/1955, 1960/1965, 1970/1975, 1976–1979, 1993–1994, and 2005–2006). Data on the population at risk (i.e., city residents < 16 years) during each period was retrieved through official records [19]. Age- and gender-standardized rates were calculated through direct standardization, with the average pediatric city population (in 1-year classes) during the study period as reference. Results are presented as number of fractures, mean fracture incidences per 100,000 person years, and as proportions (%) of all fractures. To estimate differences in rates between evaluated periods, we calculated rate ratios (RR) by use of the chi-square distribution, including 95% confidence intervals (95% CI) to describe uncertainty. We considered *p* < 0.05 as a statistically significant difference.

## Results

### All hand fractures 2005–2006

**Fig. 1:**
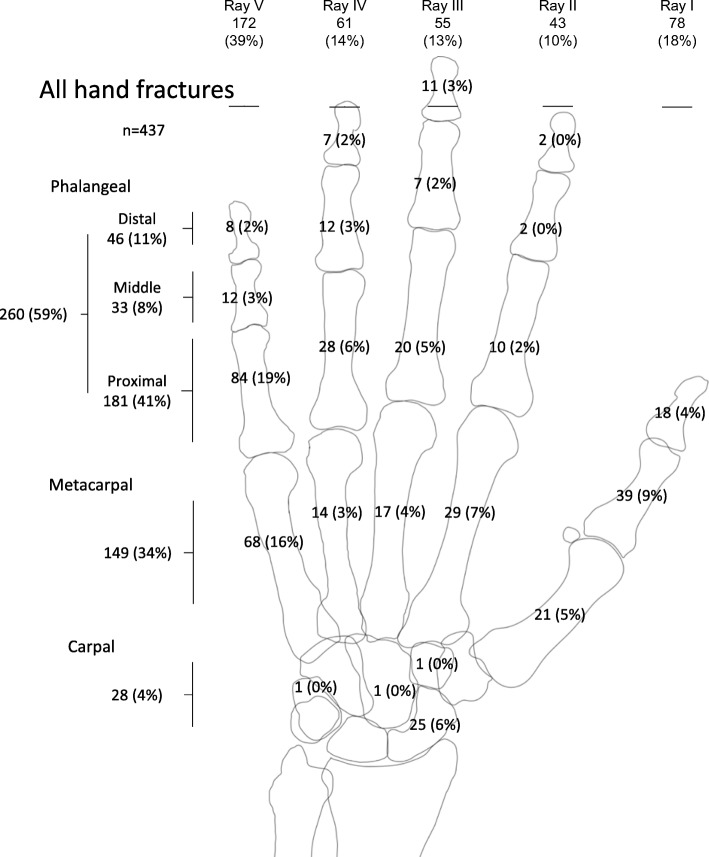
The anatomical distribution of hand fractures in individuals aged < 16 during 2005–2006, presented as number of fractures with proportion of all hand fractures in brackets. The sums for each ray 1 to 5 are presented on the top row and the sums of distal, intermediary and proximal phalangeal fractures, metacarpal fractures, and carpal fractures on the left

The distribution of hand fractures (*n* = 437) in city residents aged < 16 during 2005–2006 is reported by anatomical location in Fig. 1, by sex in Additional file 1: Figure S1, and by side in Additional file 2: Figure S2.

### Fractures 2005–2006, classified as in the previous reports from the same city [6, 9])

By this classification system (multiple fractures in phalangeal, metacarpal, and carpal bones at the same event classified as a single fracture), we found 414 hand fractures (303 in 294 boys and 111 in 108 girls). This corresponds to 24% (27% in boys and 19% in girls) of the total number of 1692 fractures (1119 fractures in 1062 boys and 573 in 553 girls) (Table 1). The crude incidence rate of hand fractures was 448/100,000 person years (639 in boys and 247 in girls), with boys having 2.5 times higher age-adjusted incidence than girls (RR 2.5; 95% CI 1.8 to 3.5) (Table 1).

**Table 1:** Number of hand fractures, crude, and age-adjusted incidence rates (/100,000 person years) in boys, girls, and all children aged < 16 during six separate periods from 1950/1955 to 2005–2006

Hand fractures in children aged < 16 in our city year 1950/1955 to 2005–2006							
		1950/1955	1960/1965	1970/1975	1976–1979	1993–1994	2005–2006
Number of fractures	All children	191	298	411	921	369	414
	Boys	131	198	269	616	250	303
	Girls	60	100	142	305	119	111
Crude incidence	All children	205	288	429	573	449	448
	Boys	275	373	549	747	591	639
	Girls	131	198	304	389	298	247
Age-adjusted incidence	All children	220	281	421	528	492	439
	Boys	297	363	537	686	653	619
	Girls	139	195	299	362	323	249

The numbers and the crude and age-adjusted incidence rates of phalangeal, carpal/metacarpal, and of scaphoid fractures are presented in Additional file 3: Table S1, Additional file 4: Table S2, and Additional file 5: Table S3. Boys compared to girls had a 70% higher age-adjusted incidence of phalangeal fractures (RR 1.7; 95% CI 1.3 to 2.2), eight times higher incidence of carpal/metacarpal fractures (RR 7.9; 95% CI 4.6 to 14.4), and 2.3 times higher incidence of scaphoid fractures (RR 2.3; 95% CI 0.6 to 36.2).

### Peak incidence rate in boys and girls in 2005–2006

**Fig. 2:**
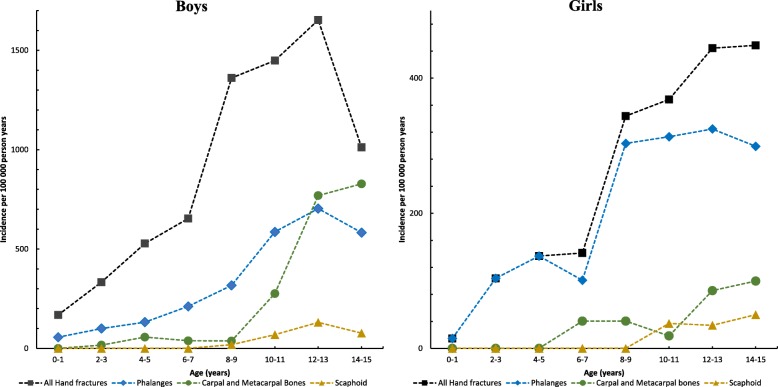
Age-specific incidence rates of all types of hand fractures, fractures of the phalanges, fractures of the metacarpals/carpal bones, and fractures of the scaphoid bone in boys and girls during 2005–2006, presented as number of fractures /100,000 person years

Boys reached the highest age-specific incidence rate for hand fractures (any), for phalangeal fractures, and for scaphoid fractures at age 12–13 years and for metacarpal/carpal fractures at age 14–15 years (Fig. 2). Girls reached the highest age-specific incidence rate for hand fractures (any) and scaphoid fractures at age 14–15 years and for metacarpal/carpal and phalangeal fractures at age 12–13 years (Fig 2).

### Side preponderance 2005–2006

We found no side preponderance for hand fractures (any) (right to left rate ratio RR 1.2; 95% CI 0.99 to 1.5) or in phalangeal fractures (RR 1.0; 95% CI 0.7 to 1.2) while metacarpal/carpal fractures were more common in the right hand (RR 2.1; 95% CI 1.5 to 3.0). Right to left rate ratios for any hand fracture, for phalangeal fractures, and for metacarpal/carpal fractures in all children, in boys, and in girls are presented in Additional file 6: Table S4.

### Time trends in incidence rates from 1950/1955 to 2005–2006

**Fig. 3:**
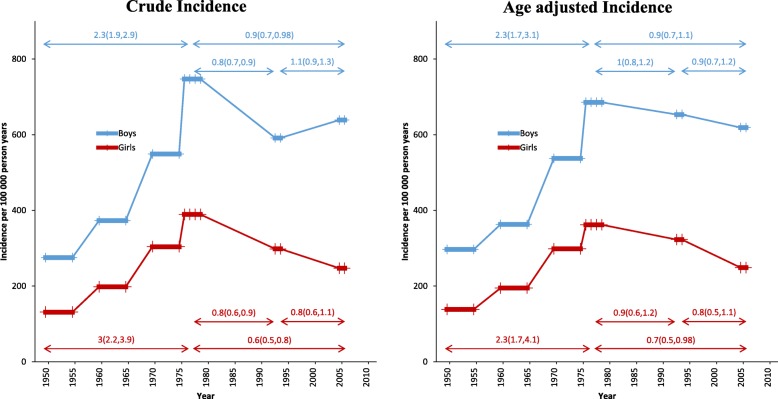
Crude and age-adjusted gender-specific incidence rates of hand fractures during six evaluated periods from 1950/1955 to 2005–2006, presented as number of fractures /100,000 person years. The included periods are indicated with horizontal thick lines from period start to period end while the evaluated years are indicated by thin crosses. Comparisons between different periods are provided as rate ratios (RR) with 95% confidence intervals (95% CI). Horizontal arrows above and below the RR indicate the compared periods

The lowest age-adjusted incidence rate of pediatric hand fractures was found in 1950/1955 and the highest in 1976–1979 (Table 1). The age- and gender-standardized fracture rate in 1976–1979 was more than twice as high as 1950/1955 (RR 2.4; 95% CI 1.9 to 3.1) (Tables 1 and 2). The age- and gender-standardized fracture rate in 2005–2006 was 20% lower than 1976/1979 (RR 0.8; 95% CI 0.7 to 1.01) but double compared to 1950/1955 (RR 2.0; 95% CI 1.6 to 2.6) (Tables 1 and 2). Gender-specific time trend data are presented in Fig. 3 and Table 2.

**Table 2:** Differences in crude and age-adjusted hand fracture incidence in children, in boys and in girls aged < 16, from 1950/1955 to 1976–1979 (previously only reported as crude changes [6]) and to 2005–2006 (changes from the first to the most recent evaluated period), from 1976/1979 to 1993/1994 (previously only reported as crude changes [9]) and to 2005–2006 (changes from the period with the highest reported fracture incidence [6] to the most recent evaluated period), and from 1993 to 1994 to 2005–2006 (changes from the last reported fracture incidence [9] to the most recent evaluated period)

Denominator		1950/1955		1976–1979		1993–1994
Nominator		1976–1979	2005–2006	1993–1994	2005–2006	2005–2006
Unadjusted	All Children	*2.8* (*2.4 to 3.3*)	*2.2* (*1.8 to 2.6*)	*0.8* (*0.7 to 0.9*)	*0.8* (*0.7 to 0.9*)	1.0 (0.9 to 1.1)
	Boys	*2.3* (*1.9 to 2.9*)	*2.3* (*1.9 to 2.9*)	*0.8* (*0.7 to 0.9*)	*0.9* (*0.7 to 0.98*)	1.1 (0.9 to 1.3)
	Girls	*3* (*2.2 to 3.9*)	*1.9* (*1.4 to 2.6*)	*0.8* (*0.6 to 0.9*)	*0.6* (*0.5 to 0.8*)	0.8 (0.6 to 1.1)
Age-adjusted	All children	*2.4* (*1.9 to 3.1*)	*2* (*1.6 to 2.6*)	0.9 (0.8 to 1.1)	0.8 (0.7 to 1.01)	0.9 (0.7 to 1.1)
	Boys	*2.3* (*1.7 to 3.1*)	*2.1* (*1.5 to 2.8*)	1.0 (0.8 to 1.2)	0.9 (0.7 to 1.1)	0.9 (0.7 to 1.2)
	Girls	*2.6* (*1.7 to 4.1*)	*1.8* (*1.1 to 2.9*)	0.9 (0.6 to 1.2)	*0.7* (*0.5 to 0.98*)	0.8 (0.5 to 1.1)

Comparisons are presented as rate ratios with 95% confidence intervals (95% CI) within brackets. Statistically significant changes are in italics

Crude- and age-adjusted incidence rates of phalangeal fractures and fractures of the metacarpal/carpal bones between the different periods are reported in Additional file 7: Table S5 and Additional file 8: Table S6. Time trends were not evaluated for scaphoid fractures due to the low number of fractures.

### Time trends from 1950/1955 to 2005–2006 in peak incidence rates in boys and girls

**Fig. 4:**
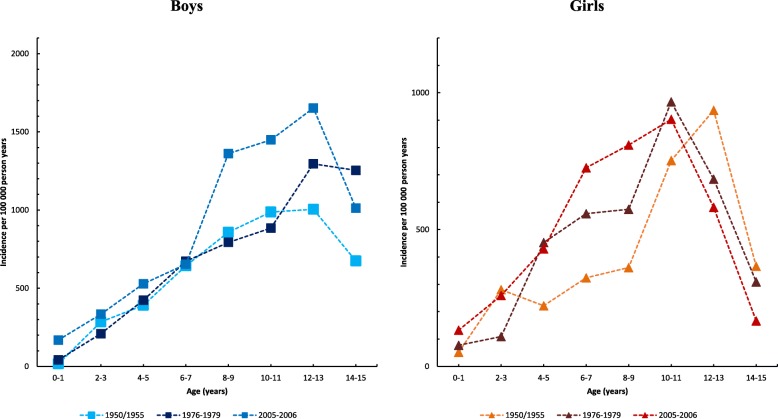
The age-specific incidence rate of hand fractures in boys and in girls during the three periods: 1950/1955 (study start), 1976–1979 (middle of study period), and 2005–2006 (study end), presented as incidences per 2-year age class /100,000 person years

Boys reached in 2005–2006 the highest age-specific hand fracture incidence at the age of 12–13 years, while the highest incidence in 1950/1955 and 1976–1979 was found at age 14–15 (Fig. 4). Girls reached in 2005–2006 the highest age-specific hand fracture incidence at age 14–15 while the highest incidence in 1950/1955 and 1976–1979 was found at age 12–13 (Fig. 4).

### Etiology of hand fractures in 2005–2006 and time trends from 1950/1955 to 2005–2006

Hand fractures etiology data are presented in Table 3, for phalangeal and metacarpal/carpal fractures in Additional file 9: Table S7 and Additional file 10: Table S8. The most common etiologies for hand fractures 2005–2006 were sports trauma, fights, and traffic accidents. The most common etiologies for hand fractures 1950/1955 were sports injuries, traffic accidents, and accidents at school. Traffic accidents and home accidents accounted for the lowest proportion of hand fractures during the evaluated years 2005–2006 while the proportion of fights as etiology was highest 2005–2006.

**Table 3:** Etiology of hand fractures in individuals aged < 16 during six separate periods from 1950/1955 to 2005–2006 (for the last period also separate in boys and girls). Etiology data was missing in 58% of cases in 1950/1955, 49% in 1960/1965, 41% in 1970/1975, 42% in 1976–1979, 28% in 1993–1994, and 30% in 2005/2006. Data are presented as the proportions (%) of different etiologies among the cases where etiology (fracture related activity) could be determined

Environmental factors	All children					All children	Boys	Girls
	1950/1955 (%)	1960/1965 (%)	1970/1975 (%)	1976–1979 (%)	1993–1994 (%)	2005–2006 (%)	2005–2006 (%)	2005–2006 (%)
Home accidents	9.9	5.9	8.7	3.2	6.8	1.0	0.5	2.7
Day nursery accidents	0	0	0.4	0.2	1.5	1.4	1.9	0
School accidents	14.8	9.8	7.0	6.2	4.5	10.4	10.7	9.6
Work accidents	3.7	0	0	0	1.5	0	0	0
Traffic accidents	21.0	16.3	14.9	14.0	18.8	13.2	13.5	12.3
Bicycle accidents	18.5	7.8	9.1	8.6	13.9	12.2	12.1	12.3
Pedestrian hit by vehicle	1.2	4.6	0.4	0.7	0	0	0	0
Moped, motorcycle	0	2.0	2.1	1.9	1.9	1.0	1.4	0
Car passenger	1.2	0.7	2.9	2.6	0.8	0	0	0
Other	0	1.3	0.4	0.2	2.3	0	0	0
Playing accidents	12.3	21.6	14.0	13.6	14.7	11.1	9.3	16.4
Playground	1.2	2.0	1.7	1.1	2.3	2.1	1.4	4.1
In-lines, skateboard	0	0	0	1.9	1.9	2.4	1.9	4.1
Sledge, other “snow”	0	0	0.8	1.7	1.1	1.0	1.4	0
Other play accidents	11.1	19.6	11.6	9.0	9.4	5.6	4.7	8.2
Sport accidents	27.2	34.0	40.1	46.7	39.8	42.4	39.1	52.1
Ball-game	19.8	26.8	30.2	33.6	26.3	29.9	28.4	34.2
Ice-hockey, skating	3.7	3.9	5.0	3.6	2.6	2.8	3.3	1.4
Gymnastics and athletics	0	0	0	1.1	2.6	0	0	0
Horse accidents	3.7	0.7	2.5	2.8	2.6	1.7	0	6.8
Wrestling, boxing, etc.	0	0.7	0.4	1.9	3.4	3.5	4.2	1.4
Skiing	0	0.7	2.1	3.4	1.5	2.1	1.9	2.7
Other	0	1.3	0	0.4	0.8	2.4	1.4	5.5
Fights	9.9	12.4	13.2	13.5	11.3	20.1	24.7	6.8
Other	1.2	0	1.7	2.6	1.1	0.3	0.5	0

## Discussion

One fourth of all pediatric fractures 2005–2006 affected the hand. Hand fractures (any) were 2.5 times and metacarpal/carpal fractures 8 times more common in boys than in girls. Hand fracture incidences increased with age in both genders until puberty. Girls reached peak incidence rates 2 years ahead of boys, that is, our data support the notion that hand fracture incidence rate is higher in boys than in girls and that there is a higher incidence with increasing age until a peak around puberty [4, 6, 9, 12, 13, 17, 23, 25, 27, 28].

The pediatric crude hand fracture incidence in 2005–2006 (448/100,000 person years) was in our city higher than 2005 in Helsinki Finland (344/100,000) [8], higher than 2006–2007 in the northern part of our country (389/100,000) [29], and 18 times higher than 1996–2001 in British Columbia (Canada) (24/100,000 person years) [24]. The discrepancies could be the result of different study methodology. For example, in our study, only one hospital serves the entire region, while several hospitals served the population at risk in the Canadian study. However, there could also be actual differences in fracture incidence rates, supported by the UK data, reporting that fracture incidences in county regions varied compared to London with a incidence rate ratio ranging from 1.0 to 1.7 [5]. Since ethnicity influences the fracture risk [15, 30, 31], a different mixture of ethnicity in different regions and countries could also explain regional fracture incidence differences.

The age-adjusted hand fracture incidence rate was in our city more than double 1976–1979 compared to 1950/1955 and 20% higher than 2005–2006 (not reaching statistical significance). This suggests that there could have been a trend break in pediatric fracture incidence at the end of the 1970s. Some of the time trends could be attributed to changes in demographics, since after adjustment for age, time trend changes were no longer statistically significant in boys. Finnish data however support a time trend change in pediatric fracture rates, reporting a higher pediatric hand fracture incidence in 2005 than in 1967 and around 50% lower incidence in 2005 than in 1983 [8]. However, in contrast to our study, the Finnish study only compared absolute incidences, but without conducting statistical comparisons.

Etiology data indicate that safety requirements for toy manufacturing, playground construction, protective sports gear, and accident prevention in traffic and home environment have positively influenced time trends in pediatric fracture incidence [32, 33]. Time trends in life style, such as more violent fights and less physical activity among children, may also influence time trends. Swedish children have reduced their everyday physical activity during recent decades, instead spending more time in front of monitors [34], and low levels of physical activity is a risk factor for fractures [35, 36]. The proportion of children with foreign background in our city was, in 2005–2006, also higher than during previous decades [37, 38], another difference that could have influenced time trends [15, 30, 31].

Study strengths include the epidemiology and etiology data from a well-defined cohort during six decades. Inclusion of only objectively verified fractures, without double counting due to multiple visits, is another strength. Weaknesses include the modification of the data collection method, due to the change in archiving method in the year 2001. However, our validation found a 3% miscalculation rate, the same as with the previous method [6, 9]. Another weakness is the risk of missing fractures exclusively treated outside the catchment area. People may be more mobile today than previously, sustaining more fractures that receive treatment at other hospitals. Since the standard treatment in Sweden still is to refer these cases to follow-up visits at the home hospitals, there is a minor risk to miss these fractures. Another confounder could be that individuals, decades ago, were less prone to seek medical advice and that doctors at that time were more hesitant to send patients to X-ray exams than today, thereby missing actual fractures. If this actually was the case is today impossible to clarify. A larger sample size would have been advantageous, especially for subgroup analyses making time trend analyses of rarer fracture types possible. Finally, the larger proportion of missing fracture etiology historically than at the last follow-up is another limitation and the reason to why we refrained from statistical analysis of time trend changes in etiology.

## Conclusions

In conclusion, the incidence rate of hand fractures is higher in older than younger children, phalangeal fractures are more common than metacarpal fractures, and hand fractures are 2.5 times more common in boys than girls. The age-adjusted incidence rate of hand fractures in children was more than doubled in 1976–1979 compared to that in 1950/1955, with obvious differences also in fracture etiology. There may have been a trend break in 1976–1979, since we found a 20% lower fracture incidence in 2005–2006 than in 1976–1979, a time trend change not reaching statistical significance but inferring the need to continue to follow pediatric fracture incidences.

## Additional files

Additional file 1: Figure S1. The anatomical distribution of hand fractures in boys and girls aged <16 during 2005-2006, presented as number of fractures with proportion of all hand fractures in the respective gender in brackets. The sums for each ray 1 to 5 are presented on the top row and the sums of distal, intermediary and proximal phalangeal fractures, metacarpal fractures and carpal fractures on the left. (PPTX 85 kb)

Additional file 2: Figure S2. The anatomical distribution of hand fractures in the left and the right hand in individuals aged <16 during 2005-2006, presented as number of fractures with proportion of all hand fractures in the respective hand in brackets. The sums for each ray 1 to 5 are presented on the top row and the sums of distal, intermediary and proximal phalangeal fractures, metacarpal fractures and carpal fractures on the left and right side, respectively. (PPTX 85 kb)

Additional file 3: **Table S1.** Number of phalangeal fractures with crude and age-adjusted incidence rates (/100,000 person years) in boys, in girls and in all children aged < 16 during six separate periods from 1950/1955 to 2005–2006. (DOCX 13 kb)

Additional file 4: **Table S2.** Number of metacarpal/carpal bones fractures (except the scaphoid bone) with crude and age-adjusted incidences (/100,000 person years) in boys, in girls and in all children aged < 16 during six separate periods from 1950/1955 to 2005–2006. (DOCX 13 kb)

Additional file 5: **Table S3.** Number of scaphoid bone fractures with crude and age-adjusted incidences (/100,000 person years) in boys, in girls and in all children aged < 16 during six separate periods from 1950/1955 to 2005–2006. (DOCX 13 kb)

Additional file 6: **Table S4.** Right to left distribution of any hand fracture, for fractures of the phalanges and the carpal/metacarpal bones (excluding the scaphoid) in all children, in boys and in girls for the period 2005–2006. Comparisons are presented as Rate Ratios (RR) with 95% Confidence Intervals (95% CI) within brackets. Statistically significant changes are bolded. The distribution of scaphoid fractures was not examined due to a low number of fractures. (DOCX 14 kb)

Additional file 7: **Table S5.** Differences in crude and age-adjusted incidence of fractures of the phalanges of the hand in children, in boys and in girls aged < 16, from 1950/1955 to 1976–1979 (previously only reported as crude changes [6]) and to 2005–2006 (changes from the first to the most recent evaluated period), from 1976/1979 to 1993/1994 (previously only reported as crude changes [9]) and to 2005–2006 (changes from the period with the highest reported fracture incidence [6] to the most recent evaluated period) and from 1993 to 1994 to 2005–2006 (changes from the last reported fracture incidence [9] to the most recent evaluated period). Comparisons are presented as Rate Ratios with 95% Confidence Intervals (95% CI) within brackets. Statistically significant changes are bolded. (DOCX 16 kb)

Additional file 8: **Table S6.** Differences in crude and age-adjusted incidence of fractures of the metacarpals/carpal bones (except the scaphoid bone) in children, in boys and in girls aged < 16. from 1950/1955 to 1976–1979 (previously only reported as crude changes [6]) and to 2005–2006 (changes from the first to the most recent evaluated period), from 1976/1979 to 1993/1994 (previously only reported as crude changes [9]) and to 2005–2006 (changes from the period with the highest reported fracture incidence [6] to the most recent evaluated period) and from 1993 to 1994 to 2005–2006 (changes from the last reported fracture incidence [9] to the most recent evaluated period). Comparisons are presented as Rate Ratios with 95% Confidence Intervals (95% CI) within brackets. Statistically significant changes are bolded. (DOCX 13 kb)

Additional file 9: **Table S7.** Etiology of phalangeal fractures in individuals aged < 16 during six separate periods from 1950/1955 to 2005–2006 (for the last period also separate in boys and girls). Etiology data was missing in 60% of cases in 1950/1955, 55% in 1960/1965, 44% in 1970/1975, 44% in 1976–1979, 31% in 1993–1994 and 31% in 2005/2006. Data are presented as the proportions (%) of different etiologies among the cases where etiology (fracture related activity) could be determined. (DOCX 15 kb)

Additional file 10: **Table S8.** Etiology of metacarpal/carpal fractures (except the scaphoid bone) in individuals aged < 16 during six separate periods from 1950/1955 to 2005–2006 (for the last period also separate in boys and girls) Etiology data was missing in 56% of cases in 1950/1955, 35% in 1960/1965, 32% in 1970/1975, 37% in 1976–1979, 22% in 1993–1994 and 33% in 2005/2006. Data are presented as the proportions (%) of different etiologies among the cases where etiology (fracture related activity) could be determined. (DOCX 15 kb)

## Data Availability

The datasets used and/or analyzed during the current study are available from the corresponding author on reasonable request.
