# Supplementary material for: Hand fracture epidemiology and etiology in children—time trends in Malmö, Sweden, during six decades
Source: J Orthop Surg Res. 2019 Jul 12;14:213. doi: 10.1186/s13018-019-1248-0 (PMC6626361; doi:10.1186/s13018-019-1248-0)
Supplement: Supplementary file 1 — The anatomical distribution of hand fractures in boys and girls aged <16 during 2005-2006, presented as number of fractures with proportion of all hand fractures in the respective gender in brackets. The sums for each ray 1 to 5 are presented on the top row and the sums of distal, intermediary and proximal phalangeal fractures, metacarpal fractures and carpal fractures on the left. (PPTX 85 kb) [file 13018_2019_1248_MOESM1_ESM.pptx]

## Slide 1
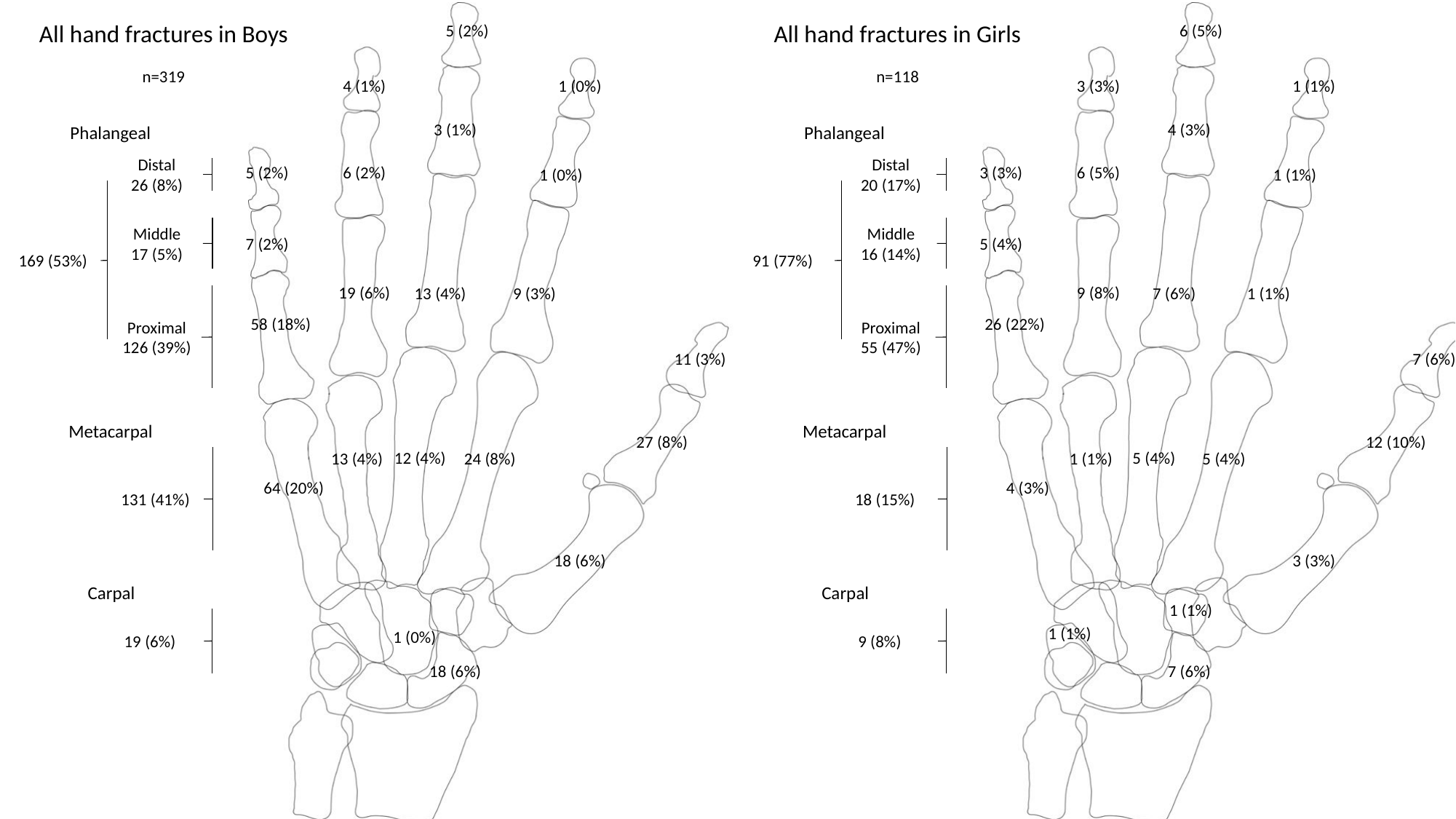

Ray III
33 (10%)
Ray II
35 (11%)
Ray IV
42 (13%)
Ray V
134 (42%)
Ray I
56 (18%)
All hand fractures in Boys
5 (2%)
n=319
4 (1%)
1 (0%)
3 (1%)
Phalangeal
5 (2%)
6 (2%)
Distal
26 (8%)
1 (0%)
169 (53%)
Middle
17 (5%)
7 (2%)
19 (6%)
13 (4%)
9 (3%)
Proximal
126 (39%)
58 (18%)
11 (3%)
Metacarpal
27 (8%)
12 (4%)
13 (4%)
24 (8%)
131 (41%)
64 (20%)
18 (6%)
Carpal
 19 (6%)
1 (0%)
18 (6%)
Ray III
22 (19%)
Ray II
8 (7%)
Ray IV
19 (16%)
Ray V
38 (32%)
Ray I
22 (19%)
All hand fractures in Girls
6 (5%)
n=118
3 (3%)
1 (1%)
4 (3%)
Phalangeal
3 (3%)
6 (5%)
Distal
20 (17%)
1 (1%)
91 (77%)
Middle
16 (14%)
5 (4%)
9 (8%)
7 (6%)
1 (1%)
Proximal
55 (47%)
26 (22%)
7 (6%)
Metacarpal
12 (10%)
5 (4%)
1 (1%)
5 (4%)
18 (15%)
4 (3%)
3 (3%)
Carpal
1 (1%)
 9 (8%)
1 (1%)
7 (6%)
