# Supplementary material for: Hand fracture epidemiology and etiology in children—time trends in Malmö, Sweden, during six decades
Source: J Orthop Surg Res. 2019 Jul 12;14:213. doi: 10.1186/s13018-019-1248-0 (PMC6626361; doi:10.1186/s13018-019-1248-0)
Supplement: Supplementary file 2 — The anatomical distribution of hand fractures in the left and the right hand in individuals aged <16 during 2005-2006, presented as number of fractures with proportion of all hand fractures in the respective hand in brackets. The sums for each ray 1 to 5 are presented on the top row and the sums of distal, intermediary and proximal phalangeal fractures, metacarpal fractures and carpal fractures on the left and right side, respectively. (PPTX 85 kb) [file 13018_2019_1248_MOESM2_ESM.pptx]

## Slide 1
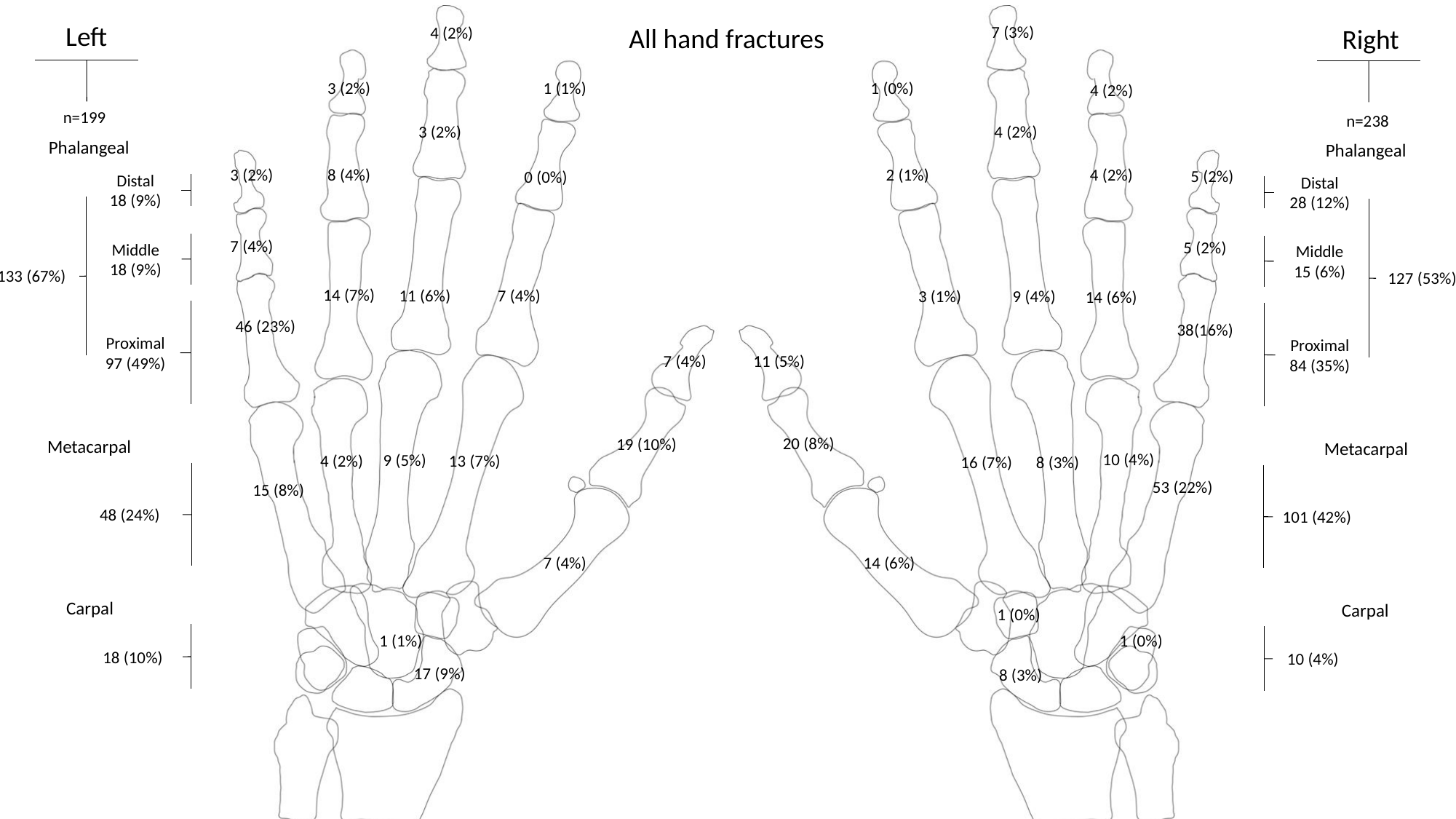

Ray IV
29 (15%)
Ray III
27 (14%)
Ray V
101 (42%)
Ray III
28 (12%)
Ray II
21 (11%)
Ray II
22 (9%)
Ray V
71 (36%)
Ray I
45 (19%)
Ray IV
32 (13%)
Ray I
33 (17%)
11
11
Left
All hand fractures
7 (3%)
Right
4 (2%)
3 (2%)
1 (1%)
1 (0%)
4 (2%)
n=199
n=238
4 (2%)
3 (2%)
Phalangeal
Phalangeal
Distal
28 (12%)
127 (53%)
Middle
15 (6%)
Proximal
84 (35%)
Metacarpal
101 (42%)
Carpal
 10 (4%)
2 (1%)
4 (2%)
3 (2%)
8 (4%)
5 (2%)
0 (0%)
Distal
18 (9%)
133 (67%)
7 (4%)
5 (2%)
Middle
18 (9%)
14 (7%)
11 (6%)
7 (4%)
3 (1%)
9 (4%)
14 (6%)
Proximal
97 (49%)
46 (23%)
38(16%)
7 (4%)
11 (5%)
20 (8%)
19 (10%)
Metacarpal
10 (4%)
9 (5%)
4 (2%)
13 (7%)
16 (7%)
8 (3%)
48 (24%)
53 (22%)
15 (8%)
14 (6%)
7 (4%)
Carpal
1 (0%)
 18 (10%)
1 (1%)
1 (0%)
17 (9%)
8 (3%)
