# Supplementary material for: Hand fracture epidemiology and etiology in children—time trends in Malmö, Sweden, during six decades
Source: J Orthop Surg Res. 2019 Jul 12;14:213. doi: 10.1186/s13018-019-1248-0 (PMC6626361; doi:10.1186/s13018-019-1248-0)
Supplement: Supplementary file 3 — Table S1. Number of phalangeal fractures with crude and age-adjusted incidence rates (/100,000 person years) in boys, in girls and in all children aged < 16 during six separate periods from 1950/1955 to 2005–2006. (DOCX 13 kb) [file 13018_2019_1248_MOESM3_ESM.docx]

Table S1. Number of phalangeal fractures with crude and age adjusted incidence rates (/100000 person years) in boys, in girls and in all children aged <16 during six separate periods from 1950/1955 to 2005-2006

| Phalangeal fractures in children aged <16 in our city year 1950/1955 to 2005-2006 | | | | | | | |
| --- | --- | --- | --- | --- | --- | --- | --- |
|  |  | 1950/1955 | 1960/1965 | 1970/1975 | 1976-1979 | 1993-1994 | 2005-2006 |
|  |  |  |  |  |  |  |  |
| Number of  fractures | All children | 122 | 202 | 280 | 641 | 245 | 248 |
|  | Boys | 75 | 124 | 156 | 370 | 151 | 160 |
|  | Girls | 47 | 78 | 124 | 271 | 94 | 88 |
|  |  |  |  |  |  |  |  |
| Crude Incidence | All children | 131 | 195 | 293 | 399 | 298 | 269 |
|  | Boys | 158 | 234 | 318 | 449 | 357 | 337 |
|  | Girls | 103 | 155 | 266 | 346 | 236 | 196 |
|  |  |  |  |  |  |  |  |
| Age adjusted Incidence | All children | 138 | 191 | 287 | 370 | 323 | 267 |
|  | Boys | 166 | 229 | 311 | 415 | 390 | 333 |
|  | Girls | 108 | 152 | 261 | 322 | 253 | 196 |
