# Supplementary material for: Hand fracture epidemiology and etiology in children—time trends in Malmö, Sweden, during six decades
Source: J Orthop Surg Res. 2019 Jul 12;14:213. doi: 10.1186/s13018-019-1248-0 (PMC6626361; doi:10.1186/s13018-019-1248-0)
Supplement: Supplementary file 4 — Table S2. Number of metacarpal/carpal bones fractures (except the scaphoid bone) with crude and age-adjusted incidences (/100,000 person years) in boys, in girls and in all children aged < 16 during six separate periods from 1950/1955 to 2005–2006. (DOCX 13 kb) [file 13018_2019_1248_MOESM4_ESM.docx]

Table S2. Number of metacarpal/carpal bones fractures (except the scaphoid bone) with crude and age adjusted incidences (/100000 person years) in boys, in girls and in all children aged <16 during six separate periods from 1950/1955 to 2005-2006.

| Fractures of the metacarpals/carpal bones (excluding the scaphoid)  in children aged <16 in our city year 1950/1955 to 2005-2006 | | | | | | | |
| --- | --- | --- | --- | --- | --- | --- | --- |
|  |  | 1950/1955 | 1960/1965 | 1970/1975 | 1976-1979 | 1993-1994 | 2005-2006 |
|  |  |  |  |  |  |  |  |
| Number of  fractures | All children | 64 | 91 | 119 | 259 | 107 | 141 |
|  | Boys | 53 | 69 | 103 | 228 | 87 | 125 |
|  | Girls | 11 | 22 | 16 | 31 | 20 | 16 |
|  |  |  |  |  |  |  |  |
| Crude Incidence | All children | 69 | 88 | 124 | 161 | 130 | 153 |
|  | Boys | 111 | 130 | 210 | 277 | 206 | 264 |
|  | Girls | 24 | 44 | 34 | 40 | 50 | 36 |
|  |  |  |  |  |  |  |  |
| Age adjusted Incidence | All children | 76 | 85 | 122 | 147 | 145 | 145 |
|  | Boys | 124 | 125 | 206 | 251 | 231 | 250 |
|  | Girls | 24 | 44 | 34 | 40 | 50 | 36 |
