# Supplementary material for: Hand fracture epidemiology and etiology in children—time trends in Malmö, Sweden, during six decades
Source: J Orthop Surg Res. 2019 Jul 12;14:213. doi: 10.1186/s13018-019-1248-0 (PMC6626361; doi:10.1186/s13018-019-1248-0)
Supplement: Supplementary file 5 — Table S3. Number of scaphoid bone fractures with crude and age-adjusted incidences (/100,000 person years) in boys, in girls and in all children aged < 16 during six separate periods from 1950/1955 to 2005–2006. (DOCX 13 kb) [file 13018_2019_1248_MOESM5_ESM.docx]

Table S3 Number of scaphoid bone fractures with crude and age adjusted incidences (/100000 person years) in boys, in girls and in all children aged <16 during six separate periods from 1950/1955 to 2005-2006.

| Fractures of the Scaphoid in children aged <16 in our city year 1950/1955 to 2005-2006 | | | | | | | |
| --- | --- | --- | --- | --- | --- | --- | --- |
|  |  | 1950/1955 | 1960/1965 | 1970/1975 | 1976-1979 | 1993-1994 | 2005-2006 |
|  |  |  |  |  |  |  |  |
| Number of  fractures | All children | 5 | 5 | 12 | 21 | 17 | 25 |
|  | Boys | 3 | 5 | 10 | 18 | 12 | 18 |
|  | Girls | 2 | 0 | 2 | 3 | 5 | 7 |
|  |  |  |  |  |  |  |  |
| Crude Incidence | All children | 5 | 5 | 13 | 13 | 21 | 27 |
|  | Boys | 6 | 9 | 20 | 22 | 28 | 38 |
|  | Girls | 4 | 0 | 4 | 4 | 13 | 16 |
|  |  |  |  |  |  |  |  |
| Age adjusted Incidence | All children | 6 | 5 | 12 | 12 | 24 | 26 |
|  | Boys | 6 | 9 | 20 | 19 | 33 | 36 |
|  | Girls | 5 | 0 | 4 | 3 | 15 | 16 |
