# Supplementary material for: Hand fracture epidemiology and etiology in children—time trends in Malmö, Sweden, during six decades
Source: J Orthop Surg Res. 2019 Jul 12;14:213. doi: 10.1186/s13018-019-1248-0 (PMC6626361; doi:10.1186/s13018-019-1248-0)
Supplement: Supplementary file 6 — Table S4. Right to left distribution of any hand fracture, for fractures of the phalanges and the carpal/metacarpal bones (excluding the scaphoid) in all children, in boys and in girls for the period 2005–2006. Comparisons are presented as Rate Ratios (RR) with 95% Confidence Intervals (95% CI) within brackets. Statistically significant changes are bolded. The distribution of scaphoid fractures was not examined due to a low number of fractures. (DOCX 14 kb) [file 13018_2019_1248_MOESM6_ESM.docx]

Table S4**.** Right to left distribution of any hand fracture, for fractures of the phalanges and the carpal/metacarpal bones (excluding the scaphoid) in all children, in boys and in girls for the period 2005-2006. Comparisons are presented as Rate Ratios (RR) with 95% Confidence Intervals (95% CI) within brackets. Statistically significant changes are bolded. The distribution of scaphoid fractures was not examined due to a low number of fractures.

|  | All Children | Boys | Girls |
| --- | --- | --- | --- |
| All Hand fractures | 1.2 (0.99 to 1.5) | **1.4 (1.1 to 1.8)** | 0.8 (0.6 to 1.2) |
| Phalanges | 1.0 (0.8 to 1.3) | 1.1 (0,8 to 1,5) | 0.8 (0.5 to 1.2) |
| Metacarpal/carpal bones | **2.1 (1.5 to 3.1)** | **2.3 (1.6 to 3.4)** | 1.3 (0.4 to 4.1) |
