# Supplementary material for: Hand fracture epidemiology and etiology in children—time trends in Malmö, Sweden, during six decades
Source: J Orthop Surg Res. 2019 Jul 12;14:213. doi: 10.1186/s13018-019-1248-0 (PMC6626361; doi:10.1186/s13018-019-1248-0)
Supplement: Supplementary file 7 — Table S5. Differences in crude and age-adjusted incidence of fractures of the phalanges of the hand in children, in boys and in girls aged < 16, from 1950/1955 to 1976–1979 (previously only reported as crude changes [6]) and to 2005–2006 (changes from the first to the most recent evaluated period), from 1976/1979 to 1993/1994 (previously only reported as crude changes [9]) and to 2005–2006 (changes from the period with the highest reported fracture incidence [6] to the most recent evaluated period) and from 1993 to 1994 to 2005–2006 (changes from the last reported fracture incidence [9] to the most recent evaluated period). Comparisons are presented as Rate Ratios with 95% Confidence Intervals (95% CI) within brackets. Statistically significant changes are bolded. (DOCX 16 kb) [file 13018_2019_1248_MOESM7_ESM.docx]

Table S5. Differences in crude and age adjusted incidence of fractures of the phalanges of the hand in children, in boys and in girls aged <16, from 1950/1955 to 1976-1979 (previously only reported as crude changes [6]) and to 2005-2006 (changes from the first to the most recent evaluated period), from 1976/1979 to 1993/1994 (previously only reported as crude changes [9]) and to 2005-2006 (changes from the period with the highest reported fracture incidence [6] to the most recent evaluated period) and from 1993-1994 to 2005-2006 (changes from the last reported fracture incidence [9] to the most recent evaluated period). Comparisons are presented as Rate Ratios with 95% Confidence Intervals (95% CI) within brackets. Statistically significant changes are bolded.

| *Denominator* | | 1950/1955 | |  | 1976-1979 | |  | 1993-1994 |
| --- | --- | --- | --- | --- | --- | --- | --- | --- |
| *Nominator* | | 1976-1979 | 2005-2006 |  | 1993-1994 | 2005-2006 |  | 2005-2006 |
|  |  |  |  |  |  |  |  |  |
| Unadjusted | All Children | **3.0 (2.5 to 3.7)** | **2.1 (1.6 to 2.6)** |  | **0.7 (0.6 to 0.9)** | **0.7 (0.6 to 0.8)** |  | 0.9 (0.8 to 1.1) |
|  |  |  |  |  |  |  |  |  |
|  | Boys | **2.1 (1.6 to 2.8)** | **2.1 (1.6 to 2.8)** |  | **0.8 (0.7 to 0.96)** | **0.7 (0.6 to 0.9)** |  | 0.9 (0.8 to 1.2) |
|  |  |  |  |  |  |  |  |  |
|  | Girls | **3.4 (2.5 to 4.6)** | **1.9 (1.3 to 2.7)** |  | **0.7 (0.5 to 0.9)** | **0.6 (0.4 to 0.7)** |  | 0.8 (0.6 to 1.1) |
|  |  |  |  |  |  |  |  |  |
|  |  |  |  |  |  |  |  |  |
| Age adjusted | All Children | **2.7 (2 to 3.6)** | **1.9 (1.4 to 2.7)** |  | 0.9 (0.7 to 1.1) | **0.7 (0.6 to 0.9)** |  | 0.8 (0.6 to 1.1) |
|  |  |  |  |  |  |  |  |  |
|  | Boys | **2.5 (1.7 to 3.7)** | **2.0 (1.3 to 3.0)** |  | 0.9 (0.7 to 1.3) | 0.8 (0.6 to 1.1) |  | 0.8 (0.6 to 1.2) |
|  |  |  |  |  |  |  |  |  |
|  | Girls | **3.0 (1.8 to 5)** | **1.8 (1.1 to 3.2)** |  | 0.8 (0.5 to 1.1) | **0.6 (0.4 to 0.9)** |  | 0.8 (0.5 to 1.2) |
|  |  |  |  |  |  |  |  |  |
