# Supplementary material for: Hand fracture epidemiology and etiology in children—time trends in Malmö, Sweden, during six decades
Source: J Orthop Surg Res. 2019 Jul 12;14:213. doi: 10.1186/s13018-019-1248-0 (PMC6626361; doi:10.1186/s13018-019-1248-0)
Supplement: Supplementary file 8 — Table S6. Differences in crude and age-adjusted incidence of fractures of the metacarpals/carpal bones (except the scaphoid bone) in children, in boys and in girls aged < 16. from 1950/1955 to 1976–1979 (previously only reported as crude changes [6]) and to 2005–2006 (changes from the first to the most recent evaluated period), from 1976/1979 to 1993/1994 (previously only reported as crude changes [9]) and to 2005–2006 (changes from the period with the highest reported fracture incidence [6] to the most recent evaluated period) and from 1993 to 1994 to 2005–2006 (changes from the last reported fracture incidence [9] to the most recent evaluated period). Comparisons are presented as Rate Ratios with 95% Confidence Intervals (95% CI) within brackets. Statistically significant changes are bolded. (DOCX 13 kb) [file 13018_2019_1248_MOESM8_ESM.docx]

Table S6. Differences in crude and age adjusted incidence of fractures of the metacarpals/carpal bones (except the scaphoid bone) in children, in boys and in girls aged <16. from 1950/1955 to 1976-1979 (previously only reported as crude changes [6]) and to 2005-2006 (changes from the first to the most recent evaluated period), from 1976/1979 to 1993/1994 (previously only reported as crude changes [9]) and to 2005-2006 (changes from the period with the highest reported fracture incidence [6] to the most recent evaluated period) and from 1993-1994 to 2005-2006 (changes from the last reported fracture incidence [9] to the most recent evaluated period). Comparisons are presented as Rate Ratios with 95% Confidence Intervals (95% CI) within brackets. Statistically significant changes are bolded.

| *Denominator* | | 1950/1955 | |  | 1976-1979 | |  | 1993-1994 |
| --- | --- | --- | --- | --- | --- | --- | --- | --- |
| *Nominator* | | 1976-1979 | 2005-2006 |  | 1993-1994 | 2005-2006 |  | 2005-2006 |
|  |  |  |  |  |  |  |  |  |
| Unadjusted | All Children | **2.3 (1.8 to 3.1)** | **2.2 (1.6 to 3)** |  | 0.8 (0.6 to 1.01) | 0.9 (0.8 to 1.2) |  | 1.2 (0.9 to 1.5) |
|  |  |  |  |  |  |  |  |  |
|  | Boys | **2.4 (1.7 to 3.3)** | **2.4 (1.7 to 3.3)** |  | **0.7 (0.6 to 0.95)** | 1 (0.8 to 1.2) |  | 1.3 (0.97 to 1.7) |
|  |  |  |  |  |  |  |  |  |
|  | Girls | 1.6 (0.8 to 3.7) | 1.5 (0.6 to 3.6) |  | 1.3 (0.7 to 2.3) | 0.9 (0.5 to 1.7) |  | 0.7 (0.3 to 1.4) |
|  |  |  |  |  |  |  |  |  |
|  |  |  |  |  |  |  |  |  |
| Age adjusted | All Children | **1.9 (1.2 to 3)** | **1.9 (1.2 to 3)** |  | 1 (0.7 to 1.4) | 1 (0.7 to 1.4) |  | 1 (0.7 to 1.4) |
|  |  |  |  |  |  |  |  |  |
|  | Boys | **2.0 (1.3 to 3.3)** | **2.0 (1.2 to 3.3)** |  | 0.9 (0.6 to 1.4) | 1 (0.7 to 1.5) |  | 1.1 (0.7 to 1.6) |
|  |  |  |  |  |  |  |  |  |
|  | Girls | 1.4 (0.4 to 7.6) | 1.4 (0.4 to 7.5) |  | 1.5 (0.6 to 4.9) | 1 (0.3 to 3.5) |  | 0.6 (0.2 to 1.8) |
|  |  |  |  |  |  |  |  |  |
