# Supplementary material for: Hand fracture epidemiology and etiology in children—time trends in Malmö, Sweden, during six decades
Source: J Orthop Surg Res. 2019 Jul 12;14:213. doi: 10.1186/s13018-019-1248-0 (PMC6626361; doi:10.1186/s13018-019-1248-0)
Supplement: Supplementary file 9 — Table S7. Etiology of phalangeal fractures in individuals aged < 16 during six separate periods from 1950/1955 to 2005–2006 (for the last period also separate in boys and girls). Etiology data was missing in 60% of cases in 1950/1955, 55% in 1960/1965, 44% in 1970/1975, 44% in 1976–1979, 31% in 1993–1994 and 31% in 2005/2006. Data are presented as the proportions (%) of different etiologies among the cases where etiology (fracture related activity) could be determined. (DOCX 15 kb) [file 13018_2019_1248_MOESM9_ESM.docx]

Table S7**.** Etiology of phalangeal fractures in individuals aged <16 during six separate periods from 1950/1955 to 2005-2006 (for the last period also separate in boys and girls). Etiology data was missing in 60% of cases in 1950/1955, 55% in 1960/1965, 44% in 1970/1975, 44% in 1976-1979, 31% in 1993-1994 and 31% in 2005/2006. Data are presented as the proportions (%) of different etiologies among the cases where etiology (fracture related activity) could be determined

| **Environmental factors** | | **1950/1955** | **1960/1965** | **1970/1975** | **1976-1979** | **1993-1994** |  | **2005-2006** | | |
| --- | --- | --- | --- | --- | --- | --- | --- | --- | --- | --- |
|  | | **All Children** | | | | |  | **All Children** | **Boys** | **Girls** |
| **Home accidents** |  | **12.2%** | **8.8%** | **12.0%** | **3.6%** | **7.1%** |  | **1.8%** | **0.9%** | **3.6%** |
| **Day nursery accidents** |  | **0.0%** | **0.0%** | **0.0%** | **0.3%** | **2.4%** |  | **1.8%** | **2.6%** | **0.0%** |
| **School accidents** |  | **6.1%** | **9.9%** | **8.9%** | **6.4%** | **4.1%** |  | **9.4%** | **7.8%** | **12.7%** |
| **Work accidents** |  | **6.1%** | **0.0%** | **0.0%** | **0.0%** | **2.4%** |  | **0.0%** | **0.0%** | **0.0%** |
| **Traffic accidents** |  | **22.4%** | **13.2%** | **15.2%** | **13.3%** | **14.1%** |  | **11.2%** | **12.2%** | **9.1%** |
|  | Bicycle accidents | 18.4% | 7.7% | 8.2% | 7.7% | 9.4% |  | 11.2% | 12.2% | 9.1% |
|  | Pedestrian hit by vehicle | 2.0% | 1.1% | 0.6% | 0.8% | 0.0% |  | 0.0% | 0.0% | 0.0% |
|  | Moped, motorcycle | 0.0% | 1.1% | 1.9% | 0.8% | 1.2% |  | 0.0% | 0.0% | 0.0% |
|  | Car passenger | 2.0% | 1.1% | 4.4% | 3.6% | 0.6% |  | 0.0% | 0.0% | 0.0% |
|  | Other | 0.0% | 2.2% | 0.0% | 0.3% | 2.9% |  | 0.0% | 0.0% | 0.0% |
| **Playing accidents** |  | **14.3%** | **19.8%** | **12.7%** | **13.8%** | **13.5%** |  | **12.9%** | **11.3%** | **16.4%** |
|  | Playground | 2.0% | 2.2% | 1.3% | 1.1% | 2.9% |  | 2.9% | 1.7% | 5.5% |
|  | In-lines, skateboard | 0.0% | 0.0% | 0.0% | 1.9% | 1.8% |  | 0.6% | 0.0% | 1.8% |
|  | Sledge, other “snow” | 0.0% | 0.0% | 0.6% | 1.4% | 1.2% |  | 1.2% | 1.7% | 0.0% |
|  | Other play accidents | 12.2% | 17.6% | 10.8% | 9.4% | 7.6% |  | 8.2% | 7.8% | 9.1% |
| **Sport accidents** |  | **32.7%** | **45.1%** | **47.5%** | **57.5%** | **48.8%** |  | **52.4%** | **52.2%** | **52.7%** |
|  | Ball-game | 26.5% | 38.5% | 39.9% | 45.3% | 36.5% |  | 42.4% | 43.5% | 40.0% |
|  | Ice-hockey, skating | 2.0% | 3.3% | 4.4% | 2.2% | 0.6% |  | 2.4% | 3.5% | 0.0% |
|  | Gymnastics and athletics | 0.0% | 0.0% | 0.0% | 1.4% | 3.5% |  | 0.0% | 0.0% | 0.0% |
|  | Horse accidents | 4.1% | 1.1% | 3.2% | 2.2% | 2.9% |  | 1.8% | 0.0% | 5.5% |
|  | Wrestling, boxing, etc. | 0.0% | 0.0% | 0.0% | 2.2% | 3.5% |  | 2.9% | 3.5% | 1.8% |
|  | Skiing | 0.0% | 0.0% | 0.0% | 3.9% | 0.6% |  | 0.6% | 0.9% | 0.0% |
|  | Other | 0.0% | 2.2% | 0.0% | 0.3% | 1.2% |  | 2.4% | 0.9% | 5.5% |
| **Fights** |  | **6.1%** | **3.3%** | **2.5%** | **2.2%** | **5.9%** |  | **10.0%** | **12.2%** | **5.5%** |
| **Other** |  | **0.0%** | **0.0%** | **1.3%** | **3.0%** | **1.8%** |  | **0.6%** | **0.9%** | **0.0%** |
