# Supplementary material for: Hand fracture epidemiology and etiology in children—time trends in Malmö, Sweden, during six decades
Source: J Orthop Surg Res. 2019 Jul 12;14:213. doi: 10.1186/s13018-019-1248-0 (PMC6626361; doi:10.1186/s13018-019-1248-0)
Supplement: Supplementary file 10 — Table S8. Etiology of metacarpal/carpal fractures (except the scaphoid bone) in individuals aged < 16 during six separate periods from 1950/1955 to 2005–2006 (for the last period also separate in boys and girls) Etiology data was missing in 56% of cases in 1950/1955, 35% in 1960/1965, 32% in 1970/1975, 37% in 1976–1979, 22% in 1993–1994 and 33% in 2005/2006. Data are presented as the proportions (%) of different etiologies among the cases where etiology (fracture related activity) could be determined. (DOCX 15 kb) [file 13018_2019_1248_MOESM10_ESM.docx]

Table S8. Etiology of metacarpal/carpal fractures (except the scaphoid bone) in individuals aged <16 during six separate periods from 1950/1955 to 2005-2006 (for the last period also separate in boys and girls) Etiology data was missing in 56% of cases in 1950/1955, 35% in 1960/1965, 32% in 1970/1975, 37% in 1976-1979, 22% in 1993-1994 and 33% in 2005/2006. Data are presented as the proportions (%) of different etiologies among the cases where etiology (fracture related activity) could be determined

| **Environmental factors** | | **1950/1955** | **1960/1965** | **1970/1975** | **1976-1979** | **1993-1994** |  | **2005-2006** | | |
| --- | --- | --- | --- | --- | --- | --- | --- | --- | --- | --- |
|  | | **All Children** | | | | |  | **All Children** | **Boys** | **Girls** |
| **Home accidents** |  | **7.1%** | **1.7%** | **2.5%** | **2.4%** | **7.1%** |  | **0.0%** | **0.0%** | **0.0%** |
| **Day nursery accidents** |  | **0.0%** | **0.0%** | **1.2%** | **0.0%** | **0.0%** |  | **1.1%** | **1.2%** | **0.0%** |
| **School accidents** |  | **25.0%** | **10.2%** | **3.7%** | **5.5%** | **6.0%** |  | **12.6%** | **14.3%** | **0.0%** |
| **Work accidents** |  | **0.0%** | **0.0%** | **0.0%** | **0.0%** | **0.0%** |  | **0.0%** | **0.0%** | **0.0%** |
| **Traffic accidents** |  | **17.9%** | **20.3%** | **13.6%** | **14.0%** | **20.2%** |  | **9.5%** | **9.5%** | **9.1%** |
|  | Bicycle accidents | 17.9% | 6.8% | 9.9% | 9.8% | 15.5% |  | 8.4% | 8.3% | 9.1% |
|  | Pedestrian hit by vehicle | 0.0% | 10.2% | 0.0% | 0.6% | 0.0% |  | 0.0% | 0.0% | 0.0% |
|  | Moped, motorcycle | 0.0% | 3.4% | 2.5% | 3.0% | 2.4% |  | 1.1% | 1.2% | 0.0% |
|  | Car passenger | 0.0% | 0.0% | 0.0% | 0.6% | 1.2% |  | 0.0% | 0.0% | 0.0% |
|  | Other | 0.0% | 0.0% | 1.2% | 0.0% | 1.2% |  | 0.0% | 0.0% | 0.0% |
| **Playing accidents** |  | **10.7%** | **25.4%** | **17.3%** | **14.0%** | **17.9%** |  | **5.3%** | **4.8%** | **9.1%** |
|  | Playground | 0.0% | 1.7% | 2.5% | 1.2% | 1.2% |  | 1.1% | 1.2% | 0.0% |
|  | In-lines, skateboard | 0.0% | 0.0% | 0.0% | 1.8% | 2.4% |  | 3.2% | 2.4% | 9.1% |
|  | Sledge, other “snow” | 0.0% | 0.0% | 1.2% | 2.4% | 1.2% |  | 0.0% | 0.0% | 0.0% |
|  | Other play accidents | 10.7% | 23.7% | 13.6% | 8.5% | 13.1% |  | 1.1% | 1.2% | 0.0% |
| **Sport accidents** |  | **17.9%** | **15.3%** | **24.7%** | **23.2%** | **25.0%** |  | **29.5%** | **25.0%** | **63.6%** |
|  | Ball-game | 10.7% | 10.2% | 9.9% | 7.9% | 8.3% |  | 12.6% | 11.9% | 18.2% |
|  | Ice-hockey, skating | 3.6% | 1.7% | 6.2% | 6.7% | 7.1% |  | 3.2% | 2.4% | 9.1% |
|  | Gymnastics and athletics | 0.0% | 0.0% | 0.0% | 0.0% | 1.2% |  | 0.0% | 0.0% | 0.0% |
|  | Horse accidents | 3.6% | 0.0% | 1.2% | 4.3% | 2.4% |  | 2.1% | 0.0% | 18.2% |
|  | Wrestling, boxing, etc. | 0.0% | 1.7% | 1.2% | 1.2% | 2.4% |  | 5.3% | 6.0% | 0.0% |
|  | Skiing | 0.0% | 1.7% | 6.2% | 2.4% | 3.6% |  | 3.2% | 2.4% | 9.1% |
|  | Other | 0.0% | 0.0% | 0.0% | 0.6% | 0.0% |  | 3.2% | 2.4% | 9.1% |
| **Fights** |  | **17.9%** | **27.1%** | **34.6%** | **39.0%** | **23.8%** |  | **42.1%** | **45.2%** | **18.2%** |
| **Other** |  | **3.6%** | **0.0%** | **2.5%** | **1.8%** | **0.0%** |  | **0.0%** | **0.0%** | **0.0%** |
